# Long-term small-fiber neuropathy and pain sensitization in survivors of pediatric acute lymphoblastic leukemia after stem cell transplantation

**DOI:** 10.1007/s00432-020-03216-8

**Published:** 2020-04-28

**Authors:** Victoria Ruscher, Sascha Lieber, Jörn-Sven Kühl, Johannes Schulte, Markus Blankenburg, Tobias Reindl, Pablo Hernáiz Driever

**Affiliations:** 1 Department of Pediatric Oncology/Hematology and Stem Cell Transplantation, Charité-Universitätsmedizin Berlin, Corporate Member of Freie Universität Berlin, Humboldt-Universität Zu Berlin, and Berlin Institute of Health, Augustenburger Platz 1, 13353 Berlin, Germany; 2 Department of Pediatric Neurology, Psychosomatics and Pain Therapy, Klinikum Stuttgart–Olgahospital and Faculty of Health, Witten/Herdecke University, Witten, Germany

**Keywords:** Acute lymphoblastic leukemia, Chemotherapy-induced peripheral neuropathy, Quantitative sensory testing, Small- and large nerve fibers, Pain sensitization

## Abstract

**Purpose:**

We aimed at describing for the first time peripheral small-fiber neurotoxicity and pain sensitization in survivors of pediatric acute lymphoblastic leukemia after stem cell transplantation (SCT).

**Methods:**

In a cross-sectional, retrospective, single-center study, we assessed 25 relapse-free long-term survivors (median age at SCT: 11 ± 4.9 years; median time between SCT and testing: 8.25 years, 19 males) using a reduced version of the pediatric-modified total neuropathy score for clinical assessment and Quantitative Sensory Testing (QST). Inclusion criteria: $\ge$ 6 years old at testing, $\le$ 18 years old at time of SCT, $\ge$ 1 year between SCT and testing.

**Results:**

Nine patients (36%) had peripheral neuropathy as defined by the clinical red-pmTNS (≥ 4). The QST parameters mechanical pain sensitivity, mechanical detection threshold, thermal sensory limen, vibration detection threshold and pressure pain threshold were significantly abnormal in the survivor cohort (*p* < 0.0038). Except for one, all survivors showed at least one abnormal QST parameter. When using QST, signs of small and large fiber dysfunction were present in 22 (88%) and 17 (68%) survivors, respectively. More than half of all survivors were found to experience pathologic sensitization to pain.

**Conclusions and implications for cancer survivors:**

Survivors of pediatric acute lymphoblastic leukemia after SCT are at high risk for long-term peripheral neuropathy with a dominating small-fiber and pain sensitization pattern.

**Electronic supplementary material:**

The online version of this article (10.1007/s00432-020-03216-8) contains supplementary material, which is available to authorized users.

## Introduction

Acute lymphoblastic leukemia (ALL) is the most common malignancy in childhood (Siegel et al. (2014)) and hematopoietic stem cell transplantation (SCT), an established curative option for patients with high-risk biology or recurrent disease (Merli et al. 2019). Since survival rates after SCT consistently increased over the last decades, long-term sequelae have become a center of interest (Socie et al. 1999; Wingard et al. 2011; Gooley et al. 2010). Central and peripheral neurological complications are reported in up to 40% of patients undergoing SCT (Brabander et al. 2000).

First-line polychemotherapy of ALL includes neurotoxic components such as vincristine (VCR) and methotrexate (Board 2002; Gomber et al. 2010) and the most common adverse effect is acute chemotherapy-induced peripheral neuropathy (CIPN) affecting up to 85% of patients (Addington and Freimer 2016; Lavoie Smith et al. 2015). Despite recovery in most cases, CIPN is an important long-term sequel compromising sensory and motor function in survivors of pediatric ALL at clinically significant levels (Ness et al. 2012).

Peripheral polyneuropathy is subdivided into damage of large fibers, small fibers and a combination of both. Large-fiber neuropathy (LFN) affects Aα-and Aβ-fibers and is clinically characterized by loss of vibration perception, proprioception and motor control (Misra et al. 2008). Small-fiber neuropathy (SFN) involves the small Aδ- and C-fibers (Hovaguimian and Gibbons 2011) characterized by abnormal sensations of heat or cold, hypersensitivities to heat or cold, paresthesia, allodynia, spontaneous pain and abnormal perception of thermal stimuli or pain (Blackmore and Siddiqi 2017). Central pain sensitization is considered as increased synaptic function within the central nervous system by nociceptive inputs and a result of use-dependent plasticity of homosynaptic and predominantly heterosynaptic potentiation in the spinal cord (Woolf 2011). Inflammation or injury of peripheral nerves may contribute to peripheral pain sensitization (Bishop et al. 2010; Perl et al. 1976). More than half of the survivors of allogenic SCT report at least one chronic health condition, associated with diminished quality of life by impaired physical function (Schultz et al. 2017). Everyday impairments like sheet intolerance, burning feet or sensitive skin are claimed in 50–58.9% of patients with SFN (Bakkers et al. 2014).

Scores assessing symptoms and clinical signs in addition to Nerve Conduction Studies (NCS) are the most accurate diagnostic methods for large fiber CIPN (England et al. 2005). As NCS do not assess small-fiber function and are painful, they are inappropriate for detecting SFN in pediatric patients. Definitive diagnosis of SFN remains a challenge as skin biopsy determining intraepidermal fiber density is the current gold standard for diagnosing SFN (Devigili et al. 2008), but remains reserved for severe SFN causes due to its invasiveness and expense (Blackmore and Siddiqi 2017). Ridehalgh et al. and others examined clinical tests to determine their sensitivity to discover SFN and found that Quantitative Sensory Testing (QST) is a valid assessment to rule out SFN (Ridehalgh et al. 2018; Hansson et al. 2007; Magda et al. 2002), which is easily combined with neurological scoring for CIPN (Blackmore and Siddiqi 2017). QST is a psychophysical tool that covers almost all somatosensory aspects by investigating large- and small-fiber function. In 2006 and further approved in 2016, the German Research Network on Neuropathic Pain (DFNS) published a standardized protocol, which allows the comparison with reference values and reduces bias in children and adolescents (Rolke et al. 2006a,b; Vollert et al. 2016). Its cost-efficiency, non-invasiveness and applicability in children are substantial advantages (Lieber et al. 2018; Blankenburg et al. 2010,2012). QST detects pain sensitization as increased responses to noxious inputs, shifting the sensitivity of pain perception to activation by innocuous stimuli, continued pain after end of stimuli, expanding neuronal receptive fields and sensitizing normal tissue (Woolf 2011).

So far, no studies addressed peripheral neuropathy in survivors of pediatric ALL after SCT. We aimed at identifying (1) especially SFN besides LFN using a questionnaire for symptoms and clinical signs for the assessment of peripheral neuropathy as well as QST, (2) patients with signs of pain sensitization with QST, and (3) clinical risk factors for peripheral neuropathy. We hypothesized to reveal abnormal somatosensory function and pain sensitization in a substantial percentage of survivors of pediatric ALL after SCT.

## Patients and methods

The Ethics Committee of Charité-Universitätsmedizin Berlin approved our study (EA2/105/16) in accordance with the Declaration of Helsinki. We identified survivors from the database of our pediatric SCT program at Charité-Universitätsmedizin Berlin.

### Patients

**Fig. 1:**
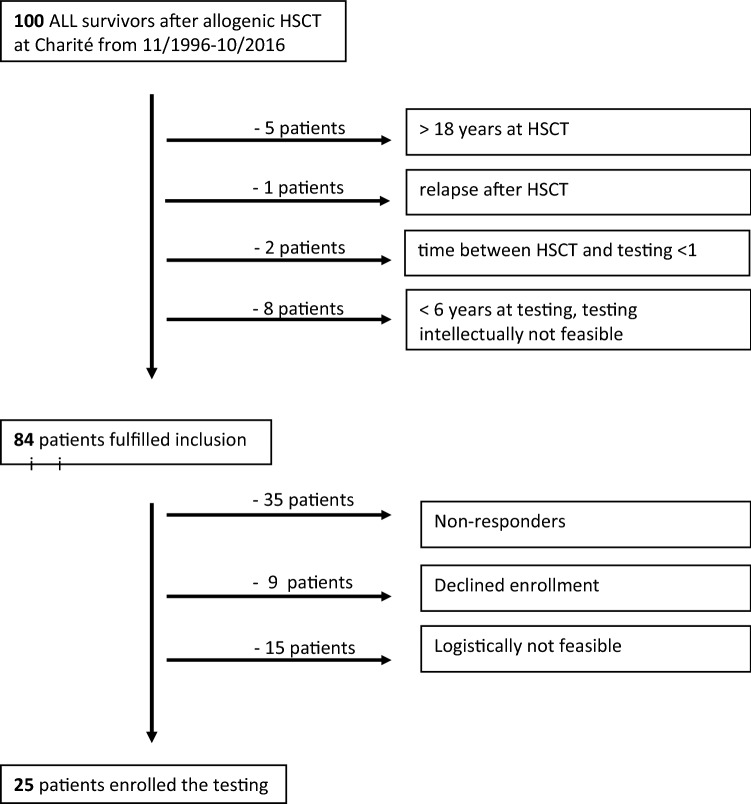
Enrollment of participants. Out of 100 ALL survivors 84 matched the inclusion criteria. Eventually, 25 survivors participated

We performed a single-center cross-sectional retrospective study with 25 of 84 eligible survivors of pediatric ALL after SCT performed between 2006 and 2016 at our center (Fig. 1). A minimum age of 6 years was obligatory for testing as both, clinical scores for CIPN and QST are validated as of this age (Blankenburg et al. 2010). Exclusion criteria for testing were age > 18 years at SCT, intellectual impairment, no relapse-free survival and < 1 year after SCT to exclude acute conditions of the treatment itself. Median recovery time between SCT and testing was 8.25 years (1.16–19 years). Patients were treated along current active protocols of first-line and SCT protocols for pediatric ALL approved by the German Society of Pediatric Oncology and Hematology (GPOH). Median cumulative vincristine dose was 12.28 mg/m^2^ (range 0–22.5 mg/m^2^). Cyclosporine A, prednisone, mycophenolate mofetil, and if needed corticosteroid pulses, were administered. Standard criteria defined acute or chronic Graft versus Host Disease (GvHD) (Filipovich et al. 2005; Martin et al. 2006, 2015). Almost all survivors had experienced acute GvHD. Grade I only affecting the skin was present in 13, grade II affecting skin and gut in 6 and grade III affecting skin, gut and liver in one survivor, respectively. Chronic GvHD was present in 3 survivors and still active during testing in one*.* Neurological complications during treatment were observed in 5 patients: Convulsions in 2, hemiplegia in one and sinus venous thrombosis in 2 patients. All these had resolved completely.

### Testing

We obtained written consent before examination and tested between 09/2016 and 04/2017 each patient for about one hour using standardized conditions as reported elsewhere (Rolke et al. 2006a).

#### Reduced pediatric-modified Total Neuropathy Score

To test for the presence of chronic pain conditions, all patients were asked to fill out the painDETECT questionnaire (Freynhagen et al. 2016) that we used in our previous study (Lieber et al. 2018). We used a reduced version of the pediatric-modified Total Neuropathy Score (red-pmTNS), consisting in 5 items (questionnaire on motor symptoms, sensory symptoms, autonomic symptoms and examination of muscle function, tendon reflexes) instead of the original 8 items to identify clinically relevant peripheral neuropathy as described elsewhere (Gilchrist and Tanner 2013). Due to double-testing, three items, light touch sensation, pin sensation and vibration, of the original pm-TNS were not included in our red-pmTNS as all 3 items were tested in QST too, but in a different approach when compared to pmTNS (Rolke et al. 2006a,b; Gilchrist and Tanner 2013; Gilchrist et al. 2014). Each category was scored from 0 to 4, with 0 given by no clinical symptoms/signs and four given by worst symptoms/signs. The scale of the red-pmTNS ranged from 0 to 20 points. Higher scores indicated worse severity of peripheral neuropathy. The cut-off score of 5 (16% of maximum score) for definition of clinical relevant peripheral neuropathy in the original pm-TNS was conservatively lowered to 4 (20% of maximum score).

#### Quantitative sensory testing

For QST, we chose the bilateral dermatomes L5/S1 on the dorsum of the feet since the longer nerve fibers on the lower limb are most likely to be impaired in peripheral neuropathy. QST was performed according to the amended DFNS protocol for children and adolescents identifying small and large fiber abnormalities and pain sensitization (Rolke et al. 2006a,b). For thermal testing, we used the TSA 2001-II (Thermal Sensory Analyzer, Medoc Ltd., Israel). In total, 13 parameters covering all somatosensory modalities were tested: Cold Detection Threshold (CDT), Warm Detection Threshold (WDT), Cold Pain Threshold (CPT), Heat Pain Threshold (HPT), Thermal Sensory Limen (TSL), Paradoxical Heat Sensations (PHS), Mechanical Detection Threshold (MDT), Mechanical Pain Threshold (MPT), Mechanical Pain Sensitivity (MPS), Wind Up Ratio (WUR), Allodynia (ALLO), Vibration Detection Threshold (VDT), Pressure Pain Threshold (PPT). According to Maier et al. we grouped QST results as combinations of losses and gains of somatosensory function (Maier et al. 2010).

### Analysis

Results of QST were analyzed according to the standardized protocol using the published reference data for gender, age and body region (Magda et al. 2002; Rolke et al. 2006a; Gilchrist and Tanner 2013) and categorized into *z* scores as described elsewhere. Statistical analysis was performed using SPSS (version 23).

## Results

### Clinical characteristics of the survivor cohort

Out of 84 eligible subjects, 25 survivors were assessed (response rate 30%). Our assessed cohort did not differ significantly from non-participants except for an increase of males and patients treated with SCT in first remission (Table 1).

**Table 1:** Characteristics of participants and non-participants

	Participants (*n* = 25)	Non-participants (*n*= 59)
Median age (range) in years	18 (8–33)	18 (3–34)
Gender		
Male	19	30
Female	6	29
Malignancy		
Precursor-B-ALL	17	46
T-ALL	6	9
Unclassified	2	4
Median age at SCT (range)	11 (2–18)	7 (1–18)
Source of stem cells		
Bone marrow	17	38
Peripheral blood stem cells	8	21
Donor		
Related	4	22
Unrelated	21	37
Stage of disease		
CR1	16	18
CR2	9	36
CR3	-	5

Ratio male: female of participants was 3.2:1 in comparison to all eligible subjects of 1.4:1. The ratio CR:CR2 of participants was 1.77:1 in comparison to all eligible subjects of 0.74:1

### Symptoms and clinical signs of peripheral neuropathy

**Fig. 2:**
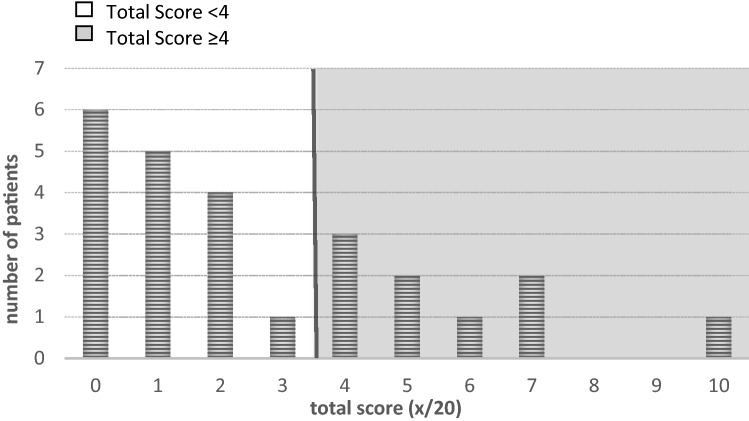
Distribution of total scores in the reduced pediatric-modified Total Neuropathy Score. Total scores of 4 or higher (grey colored background) indicated peripheral neuropathy. y-axis: number of patients; x-axis: total score in red-pmTNS

The median of the total reduced pediatric-modified Total Neuropathy Score was 2 (0–10) (Fig. 2). The median was zero for each item, except for autonomic symptoms with a median value of one. Autonomic symptoms were noted in 15 survivors (60%), 8 patients (32%) reported on sensory symptoms, and 3 (12%) on motor symptoms. Clinical signs like reduced muscle strength or loss of deep tendon reflexes were noted in 8 (32%). Nine survivors (36%) scored on or above the cut-off level of 4 in total. All of them reported autonomic symptoms; whereas, the other items were varying. No survivor reported chronic pain conditions in the painDETECT questionnaire.

### QST reveals somatosensory deficits and pain sensitization in the survivor cohort

In total, 24/25 (96%) survivors showed at least one significant abnormal parameter (i.e., z-score above or below 1.96) in QST, 17 (68%) at least two and 14 (56%) three or more parameters (Table 2). Nearly, half of the patients had increased thresholds for vibration (14 patients; 56%) and tactile detection (10 patients; 40%). One-quarter of patients had increased thermal detection thresholds for thermal distinction (7 patients, 28%) and cold (2 patients, 8%).

**Table 2:**
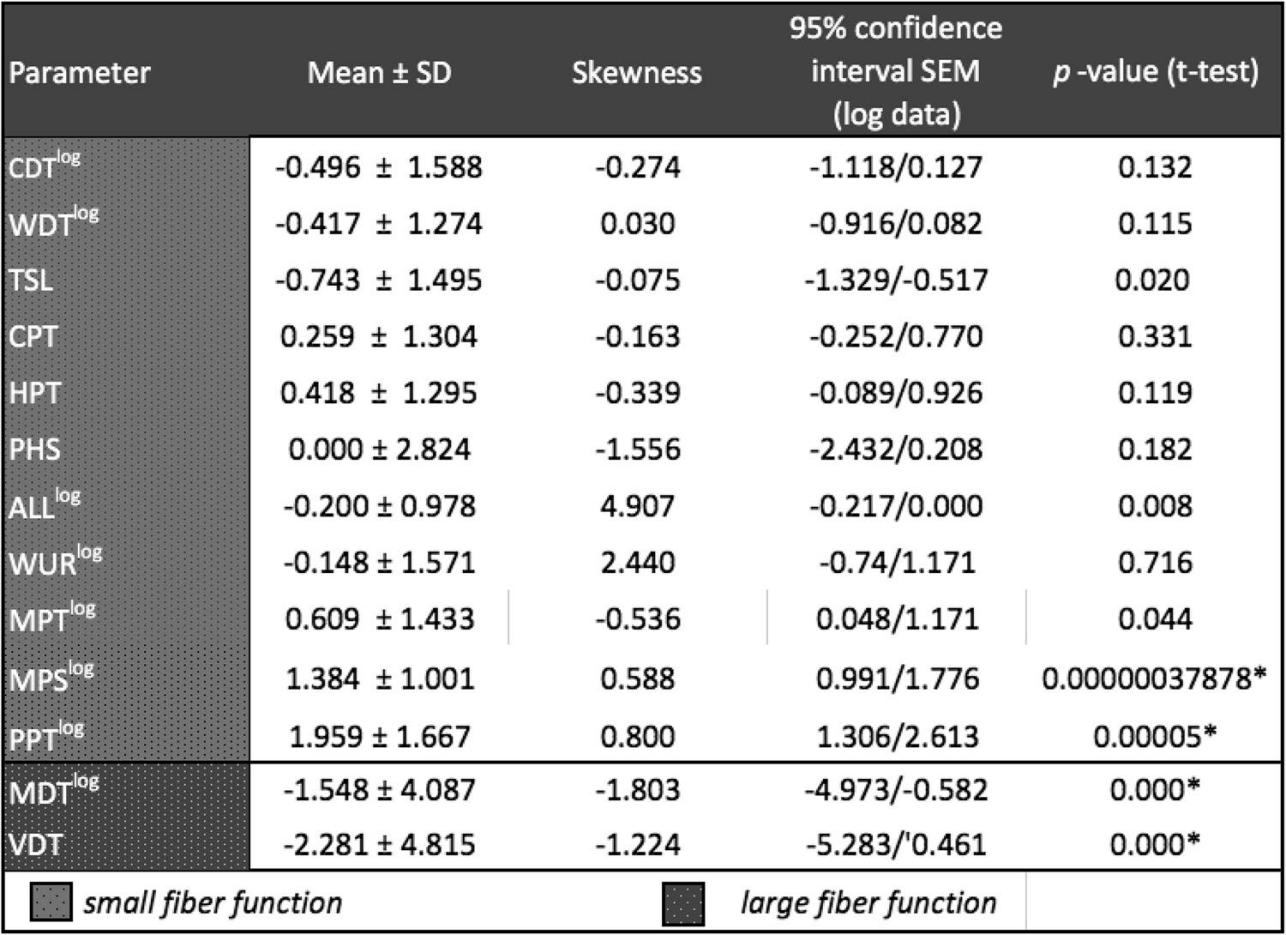
QST-results of survivors for all 13 parameters presented in Z-Scores

*p* values were analyzed with one-sided t-test for normally distributed parameters and with one-side non-parametric Wilcoxon-test for parameters with a skewness > 1 or < − 1 after transformation. We performed Bonferroni-adjustment for statistical-significance due to multiple testing and defined *p* < 0.0038 as statically significant

One-third of survivors had decreased thresholds for mechanical nociceptive stimulation regarding pressure (8 patients, 32%) and mechanical pain (6 patients, 24%). Only few survivors showed decreased nociceptive thermal perception for cold (2 patients, 8%) and warm (3 patients, 12%). Nine (32%) survivors showed paradox heat sensations (PHS). A patient was diagnosed with the phenomenon of pain sensitization when showing at least one abnormal finding such as hyperalgesia to pressure, mechanical stimuli, allodynia or when testing wind-up ratio. This meant that the perception of a stimulus that is not painful in the healthy age-matched population was already perceived as painful. Half of our cohort fulfilled this criterium (13/25), i.e., that survivors had at least one abnormal finding in the four above-mentioned tests. Among them, seven survivors showed pain sensitization in one QST parameter, five in two and one in three, respectively.

Isolated positive symptoms like hyperalgesia to thermal or mechanical stimuli, allodynia and PHS were found in 4 patients (16%). Isolated negative symptoms like hypoesthesia and hypoalgesia to thermal or mechanical stimuli were detected in 7 patients (28%). Thirteen survivors (52%) presented a combination of positive and negative symptoms. The median number of abnormalities in QST was 3 (range: 0–7, SD 1.915).

#### LoGa-Classification of the survivor cohort

When grouped into the LoGa scheme of losses and gains of function, the most frequent somatosensory deficit combination was L3G2 found in 7 patients (28%) (Table 3). Within the L3G2-combination, 5 out of 7 patients (71%) scored a total of 4 or higher in the red-pmTNS.

**Table 3:**
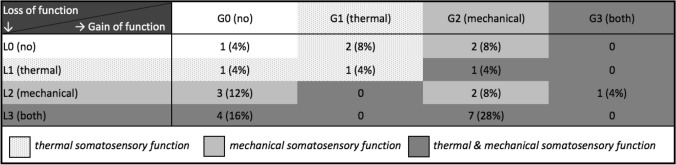
Frequency of combinations of somatosensory abnormalities

*L0* no somatosensory dysfunction; *L1* loss of thermal detection; *L2* loss of mechanical detection; *L3* loss of thermal and mechanical detection; *G0* no gain of function (hyperalgesia); *G1* thermal hyperalgesia; *G2* mechanical hyperalgesia; *G3* mixed thermal and mechanical hyperalgesia

The other way around, survivors with pathologic red-pmTNS showed a L3G2-pattern in 30%.

#### Small and large fiber neuropathy in the survivor cohort

Isolated LFN was shown in 2 patients (8%), whereas 7 patients (28%) revealed a dominant SFN. A combination of LFN and SFN was found in 15 patients (60%).

### Risk factors associated with peripheral neuropathy

We identified no significant clinical risk factor for abnormal red-pmTNS score and only one for QST parameters. Using Pearson’s correlation shorter recovery time after SCT was associated with increased sensitivity to pinprick stimuli (Pearson’s correlation coefficient: -0.573, p-value 0.003) and decreased VDT (Pearson’s correlation coefficient: 0.507, p-value 0.009).

## Discussion

The red-pmTNS uncovered signs and symptoms of functional impairment in about one out of three survivors. In 2013, Gilchrist introduced the pmTNS and demonstrated its validity and reliability in pediatric cancer patients (Gilchrist and Tanner 2013). In total, 86% and 68% displayed signs and symptoms of CIPN using the pmTNS and the CTCAE criteria at the end of therapy, respectively. When using the CTCAE criteria, about 40% of the children with pathological scores of 5 or higher in the pmTNS were missed (Lavoie Smith et al. 2015; Gilchrist et al. 2014). In our previous study in survivors after pediatric ALL treated with chemotherapy alone (median recovery time 2.5 years), 33% of survivors showed pathological results in red-pmTNS (Lieber et al. 2018) when compared to 36% of the survivors in our cohort (median recovery time 8.25 years). We suggest that SCT does not add clinical signs of PN investigated by scoring systems like red-pmTNS. Genetic variations, i.e., in CYP3A5, may change VCR metabolisms and, therefore, critically influence CIPN development (Egbelakin et al. 2011). Similarly, previous studies using comparable clinical CIPN scores in childhood ALL survivors also identified 27–41% showing peripheral neuropathy (Tay et al. 2017; Jain et al. 2014; Varedi et al. 2018a) and are in line with our observations. On the contrary, Ramchandren et al. uncovered clinical signs of peripheral neuropathy in all survivors 7.4 years after end of therapy using the Neuropathy Impairment Scale (NIS) (Tay et al. 2017). The prevailing motor function within NIS when compared to red-pmTNS may explain the discrepancy. In a longitudinal study, Gilchrist et al. showed high recovery rates with abnormal pmTNS ratings of 85% during treatment and 12%, six months after chemotherapy (Gilchrist et al. 2017). In a long-term follow-up of childhood ALL survivors (median time of 29.9 years since diagnosis), Ness et al. outlined long-lasting negative impacts of antileukemic treatment on neuromuscular function in almost 50% of survivors (Ness et al. 2012): Patients performed poorer in balance, 6-min walking distance, deep tendon reflexes, and vibration detection. Several studies have shown associations between higher scores in pmTNS and poorer performance in either static or dynamic balance or 6-min walking distance (Gilchrist and Tanner 2013; Varedi et al. 2018a). Further studies should investigate these associations by adding performance tests to trial scores.

Thirty-six percent of our cohort showed abnormalities in the red-pmTNS, but 96% had at least one abnormality in QST, highlighting the broad silent LFN and SFN in our survivor group when using the clinical scoring system only. The American Academy of Neurology reported a definition for diagnosing distal symmetric polyneuropathies using combinations of symptoms, clinical signs and electrodiagnostic tests (England et al. 2005; England and Asbury 2004; Gewandter et al. 2019), indicating the use of scoring systems as additional but not sole diagnostic criterion for PN (Cavaletti et al. 2010,2013; Gilchrist 2012).

QST as our main testing modality covers almost all somatosensory functions. When compared to our previous study in ALL survivors treated with chemotherapy only, similar distribution of significant pathologic QST parameters representing large- and small-fiber deficits were found, i.e., Mechanical Detection Threshold, Pressure Pain Threshold and Thermal Sensory Limen (Lieber et al. 2018). The similarity could be due to neurotoxic vincristine that leads to axonal degeneration and was used at varying cumulative doses before undergoing SCT (Gomber et al. 2010; Lavoie Smith et al. 2015; Mora et al. 2016). We detected signs of LFN in about two-thirds of ALL survivors with and without SCT (Lieber et al. 2018). In contrast, signs of SFN were discovered in 88% of the current cohort, compared to only a third ALL survivors having received chemotherapy only (Lieber et al. 2018) indicating additional damage associated with SCT. The similar cumulative doses of VCR in survivors after SCT and chemotherapy only, i.e., 12.3 and 12 mg/sqm, respectively, do not explain the difference. Bilic et al. used QST in a prospective study on adults with chronic GvHD following SCT and found an isolated SFN in 11%, isolated LFN in 15% and mixed SFN and LFN in 67% of survivors. These data corroborate our findings of neuropathy type distribution, i.e., isolated SFN in 28%, isolated LFN in 8% and a mixed SFN and LFN in 60% of survivors, although we could not find any association between GvHD and neuropathy in our study. As chronic GvHD most likely resembles an autoimmune mediated pathology plus reduced intensity conditioning in contrast to myeloablative conditioning was associated with a higher incidence of SFN and peripheral nerve damage following SCT (Hoeijmakers et al. 2012), peripheral neuropathy following SCT may be caused by immune-mediated mechanisms like altered dermal and epidermal immune cell and cytokine composition and keratinocyte activation damaging particularly small fibers (Grauer et al. 2010; Hoeijmakers et al. 2012). Furthermore, immunosuppressive drugs following SCT like Cyclosporine A or Tacrolimus may cause additional nerve damage (Arnold et al. 2013).

Interestingly, QST parameters reflecting pain sensitization like MPS, WUR and ALLO were more pronounced with 50% in the current cohort compared to 30% of ALL survivors with chemotherapy only. Among them, seven showed pain sensitization in one QST parameter, five in two and one in three, respectively. Most importantly, pain sensitization does not imply a chronic pain condition by itself. According to Woolf et al. repetitive Aβ-inputs can trigger hyperalgesia via conditioned C-fibers (Woolf 2011). Also, mechanical and thermal loss of detection may be compensated by reduced thresholds for other stimuli, such as pain, resulting in hyperalgesia (Baron et al. 2017; Simone et al. 1991). In our cohort, 76% of survivors showing symptoms of hyperalgesia also had signs of thermal or mechanical hypoesthesia at the same time. Still, pathomechanisms of SFN and pain sensitization are still unknown (Terkelsen et al. 2017). Previous investigations suggest that elevation of macrophages and mast cells in immune-mediated SFN leads via chemokines and other mediators to microglia cell activation (Marchand et al. 2005; Karl et al. 2019). This peripheral activation together with altered sensory stimuli processing in the CNS may contribute to pain sensitization (Hoeijmakers et al. 2012). Moreover, gene variations in SCN9A encoding Na_v_1.7 sodium channel which carries out a gain-of-function by increased spontaneous firing and sensitivity to depolarizing stimuli may contribute to additional SFN (Faber et al. 2012). Nevertheless, it is important to keep in mind that survivors were burdened with long hospital stays and may have reacted differently to the QST testing as healthy individuals due to sensitization towards the environment (Vaudre et al. 2005). Still interestingly in a study by Schultz et al., SCT survivors categorized their quality of life in a self-assessment as excellent/very good/good, although more than half of them suffered at least one chronic health conditions (Schultz et al. 2017), highlighting survivors’ changed perception of health and its links.

When categorizing deficit patterns of our current cohort as well as ALL survivors with chemotherapy only into the LoGa-classification, the most frequent somatosensory deficit combinations were L3G2 and L3G0. This is in line with Maier et al. who found patients with central pain sensitization, peripheral neuropathy and peripheral nerve injury most frequently displayed a L3G2 and L3G0 pattern (Maier et al. 2010). The similarity of LoGa-patterns may indicate the same principal underlying cause for somatosensory deficits, i.e., chemotherapy. Still, several studies showed the multifactorial genesis of CIPN, including genetic factors like CYP3A5 metabolism, treatment-related factors like drug concentration, and concomitant treatment with interacting medications like azoles (Egbelakin et al. 2011; Mora et al. 2016; Velde et al. 2017), outlining the difficulty to elucidate CIPN’s underlying pathomechanisms. Among the most affected survivors (L3G2 pattern), almost all reported difficulties in daily living concerning difficulties with zipping zippers, tripping more often, going up the stairs or decreased strength, which underlines the impact on daily living.

Three survivors after chemotherapy only reported chronic pain (Lieber et al. 2018), whereas all survivors after SCT negated it. As our cohort of survivors showed considerably more SFN and pain sensitization, we expected different results. Bakkers et al. showed that 60% of SFN patients suffer burning feet symptoms at least occasionally (Bakkers et al. 2014). Our small sample size of *n* = 25 may cause our controversial results.

We found a negative correlation between recovery time and severity of hyperalgesia for pinprick stimuli as well as hypoesthesia for vibration. Few studies support our findings of improvement in peripheral nerve function and chronic pain conditions over time following ALL treatment (Jain et al. 2014; Ramchandren et al. 2009; Miltenburg and Boogerd 2014), and yet impairment in peripheral nerve function and chronic pain conditions may still be present years after treatment (Lavoie Smith et al. 2015; Mora et al. 2016; Ramchandren et al. 2009). Treatment options for pediatric cancer survivors suffering from long-term CIPN are understudied. Data on gabapentin and glutamic acids as protective agents against VCR toxicity are inconsistent (Kandula et al. 2016; Anghelescu et al. 2011; Rao et al. 2007).

QST as a psychophysical assessment tool is prone to errors, especially in children as testing lasts for about one hour and attention and collaboration are essential (Rolke et al. 2006a). The participation rate of only 30% of eligible survivors and the shifted gender and clinical remission ratio (see *Table **1*) may reflect a selection bias. Nevertheless, age- and sex-matched reference data moderated its impact on our results. Our red-pmTNS is another limitation of our study. Still, with our study we aimed to compare our QST results with a clinical score. Survivors were examined after SCT. Hence, differentiation between CIPN prior to SCT or due to SCT is limited. (Lavoratore et al. 2009; Wei et al. 2018). Investigating survivors of SCT receiving no neurotoxic drugs before SCT, e.g., patients with metabolic disorders or hemoglobinopathies may help identifying contributing factors to small and large fiber damage in the SCT setting. So far, studies on survivors of SCT during childhood due to, i.e., sickle cell disease examined long-term central nervous system deficits, but not somatosensory functions (Walters et al. 1995,2010). Here, further longitudinal studies are needed to elucidate the contribution of SCT to long-term neuropathies, particularly SFN. The wide range of recovery time and age at testing among our patients may also affect our findings, as different developmental levels during treatment and at testing may influence this psychophysical assessment (Hirschfeld et al. 2012; Blankenburg et al. 2011). QoL may have given more insights into the clinical relevance of our study (Vetsch et al. 2018; Corella Aznar et al. 2019; Ness et al. 2015; Goebel et al. 2019), but was not measured due to already extensive testing. In future studies, large- and small-fiber neuropathy leading to motor function impairment could be tested by, e.g., 6-min walking test, and timed up-and-go test (Varedi et al. 2018b).

In conclusion, we firstly uncovered that survivors of pediatric acute lymphoblastic leukemia after SCT are at high risk for long-term peripheral neuropathy with a dominating small-fiber and pain sensitization pattern by applying scoring systems and QST as a reliable and valid diagnostic device bearing in mind its limitations. Mechanisms of nerve damage during antileukemic treatment and SCT as well as genetic variations influencing dimensions of sequelae should be the center of interest for future investigations to elucidate the contribution of the varying factors to CIPN in ALL survivors after SCT.

## Electronic supplementary material

Below is the link to the electronic supplementary material.

Supplementary file1 (DOCX 15 kb)

## Abbreviations

ALL: Acute lymphoblastic leukemia
Allo: Allodynia
CDT: Cold detection threshold
CIPN: Chemotherapy-induced peripheral neuropathy
CPT: Cold pain threshold
GvHD: Graft versus host disease
HPT: Heat pain threshold
DFGN: German Research Network on Neuropathic Pain
LFN: Large-fiber neuropathy
MDT: Mechanical detection threshold
MPT: Mechanical pain threshold
MPS: Mechanical pain sensitivity
NCS: Nerve conduction studies
QST: Quantitative sensory testing
pm-TNS: Pediatric-modified Total Neuropathy Score
PHS: Paradoxical heat sensations
PPT: Pressure pain threshold
red-pmTNS: Reduced pediatric-modified Total Neuropathy Score
SD: Standard deviation
SFN: Small-fiber neuropathy
TSL: Thermal sensory limen
SCT: Stem cell transplantation
VCR: Vincristine
VDT: Vibration detection threshold
WDT: Warm detection threshold
WUR: Wind-up-ratio
