# Supplementary material for: Long-term small-fiber neuropathy and pain sensitization in survivors of pediatric acute lymphoblastic leukemia after stem cell transplantation
Source: J Cancer Res Clin Oncol. 2020 Apr 28;146(8):2143–52. doi: 10.1007/s00432-020-03216-8 (PMC8363542; doi:10.1007/s00432-020-03216-8)
Supplement: Supplementary file 1 — Supplementary file1 (DOCX 15 kb) [file 432_2020_3216_MOESM1_ESM.docx]

**Reduced pediatric-modified total neurpathy score (red-pmTNS)**

**QUESTIONNAIRE**

Sensory symptoms

„Do you have any parts of your body that are tingly, numb (can hardly feel),

or hurt? “

0 = none

1 = symptoms limited to fingers or toes

2 = symptoms extend to ankles or wrists

3 = symptoms extend to knee or elbow

4 = symptoms above knee or elbow

🡪 record worst score for the three sensations

Functional symptoms

“Do you have trouble buttoning shirts or zipping zippers?”

“Do you have trouble walking such as tripping frequently?”

“Do you have trouble going up or down stairs?”

Record after each question: It is…

0 = not difficult

1 = a little difficult

2 = somewhat difficult

3 = I need help

4 = I can’t do that at all

🡪 record worst score of the three symptoms

Autonomic symptoms

“Do you feel dizzy or lightheaded when you get up out of bed?”

“Do your hands or feet feel hotter or colder than normal?”

Record after each question: It is…

0 = never

1 = a little bit

2 = sometimes

3 = very much

4 = almost always

🡪 record worst score for the tw sensations

**CLINICAL TESTING**

Strength

Tested body parts: great toe extension, foot dorsal extension (ankle extension), finger abduction, hand extension (wrist extension)

0 = normal

1 = mild weakness

2 = moderate weakness

3 = severe weakness

🡪 record worst score for strength

Reflexes

Tested reflex zones: Achilles reflex, patellar reflex

0 = normal

1 = ankle reflex reduced

2 = ankle reflex absent (achilles 0, patellar +2)

3 = ankle reflex, patellar reduced (Achilles 0, patellar +1)

4 = all reflexes absent

🡪 record worst score for reflexes

TOTAL SCORE: __ / 20
